# Initial experiences of robotic versus conventional laparoscopic surgery for colorectal cancer, focusing on short-term outcomes: a matched case-control study

**DOI:** 10.1186/s12957-015-0517-6

**Published:** 2015-03-12

**Authors:** Hiroyuki Sawada, Hiroyuki Egi, Minoru Hattori, Takahisa Suzuki, Manabu Shimomura, Kazuaki Tanabe, Masazumi Okajima, Hideki Ohdan

**Affiliations:** Department of Gastroenterological and Transplant Surgery, Applied Life Sciences, Institute of Biomedical & Health Sciences, Hiroshima University, 1-2-3 Kasumi, Minami-ku, Hiroshima 734-8551 Japan; Advanced Medical Skills Training Center, Institute of Biomedical & Health Sciences, Hiroshima University, 1-2-3 Kasumi, Minami-ku, Hiroshima 734-8551 Japan; Department of Surgery, Hiroshima City Hospital, 1-2-3 Kasumi, Minami-ku, Hiroshima 734-8551 Japan

**Keywords:** Robotic surgery, Laparoscopic surgery, Colon cancer, Rectal cancer, Propensity score

## Abstract

**Background:**

Robotic surgery is a new technique with the benefits of a three-dimensional view, the ability to use multi-degree-of-freedom forceps, the elimination of physiological tremors, and a stable camera view. The aim of this study was to evaluate the feasibility and short-term outcomes of robotic surgery for colorectal cancer as initial cases, compared with conventional laparoscopic surgery.

**Methods:**

From July 2010 to June 2013, ten patients with left-sided colon and rectal cancer underwent robotic surgery, and 121 received conventional laparoscopic surgery. Both groups were balanced in terms of age, gender, American Society of Anesthesiologists (ASA) score, body mass index (BMI), operative history, TNM staging, and tumor location. Moreover, in order to improve objectivity and approximate a randomized controlled study, we used the propensity score matching method. The matching was successful because the ROC analysis showed a well-balanced curve (*C* = 0.535).

**Results:**

Following propensity score matching, ten patients were included in the robotic surgery group and 20 patients were included in the conventional laparoscopic surgery group. There were no significant differences in the short-term clinicopathologic outcomes between the robotic surgery group and the conventional laparoscopic surgery group. However, the operative time was significantly longer in the robotic surgery group than in the conventional laparoscopic surgery group.

**Conclusions:**

There were no significant differences between the robotic surgery group and the conventional laparoscopic surgery group with respect to short-term clinicopathologic outcomes, with the exception of the operative time. Our early experience indicates that robotic surgery is a promising tool, particularly in patients with rectal cancer.

**Electronic supplementary material:**

The online version of this article (doi:10.1186/s12957-015-0517-6) contains supplementary material, which is available to authorized users.

## Background

Laparoscopic surgery for colon cancer has become an accepted standard treatment strategy in recent years not only for early colon cancer but also for advanced disease, with regard to oncological safety and feasibility [1-6]. However, there is little evidence in the literature regarding the use of laparoscopic surgery in patients with rectal cancer, and the application of the technique in this setting remains controversial. The technical feasibility of performing laparoscopic surgery in patients with rectal cancer has been demonstrated by experienced laparoscopic surgeons; however, even experts report technical difficulties due to the confined space in the pelvis and the limitations of existing laparoscopic instruments, which have a restricted range of movement compared with that of the surgeon’s hands. Moreover, previous reports have shown higher rates of conversion to open surgery, positive circumferential margins, and increased anastomotic leakage in patients treated with laparoscopic surgery [4,7-10]. The technical difficulties encountered when performing laparoscopic surgery for lower rectal cancer are primarily due to the anatomy of the pelvis, which lacks a wide space to use long, straight, and rigid laparoscopic instruments.

Robotic surgery is a new technique with the benefits of a three-dimensional view, the ability to use multi-degree-of-freedom forceps, the elimination of physiological tremors, and a stable camera view. It has been successfully applied in urologic surgery, and the anatomy of the pelvis suggests that performing robotic surgery in this setting is feasible, especially in patients undergoing rectal cancer surgery. To date, several studies have demonstrated the safety and feasibility, as well as acceptable short-term outcomes, of robotic colorectal surgery [11-14]. However, evidence supporting the use of robotic surgery in patients with colorectal cancer is limited, particularly with respect to randomized controlled studies.

The aim of this study was to evaluate the feasibility of robotic surgery compared with conventional laparoscopic surgery for colorectal cancer. We conducted a matched case-control study using the propensity score matching method to assess the short-term outcomes of robotic surgery for colorectal cancer compared with those of conventional laparoscopic surgery.

## Methods

This study was performed with permission of the Ethics Committee of the Hiroshima University.

We have maintained a prospective database of all operations for colorectal cancer performed since April 1994. Although this study was a retrospective review of data prospectively collected from our database, the propensity score matching method was employed. The inclusion criterion was the presence of left-sided colon and rectal cancer. The robotic surgery group included selected patients who completed treatment between July 2010 and June 2013 (*N* = 10). Patients who underwent conventional laparoscopic surgery for left-sided colon and rectal cancer between July 2010 and June 2013 were selected for enrollment in the control group (*N* = 121).

This was a matched case-control study using the propensity score matching method to compare the outcomes of robotic surgery and conventional laparoscopic surgery for colorectal cancer. The patients were matched with regard to age, gender, American Society of Anesthesiologists (ASA) score, body mass index (BMI), history of prior abdominal surgery, tumor location, clinical stage, and the preoperative serum CEA level. No analyses of surgical parameters or outcomes were conducted until the groups were definitively selected as the best comparison cohort based on preoperative variables only. The perioperative results included the operative time, amount of estimated blood loss, the need for open conversion or further surgery, complications, flatus passage, the length of postoperative hospital stay, and the number of retrieved lymph nodes.

### Robotic surgical technique

After obtaining informed consent, each patient with left-sided colon and rectal cancer was placed in the lithotomy position. Both robotic surgery and conventional laparoscopic surgery were performed using a medial-to-lateral approach with left colic artery-preserving lymphadenectomy.

**Figure 1:**
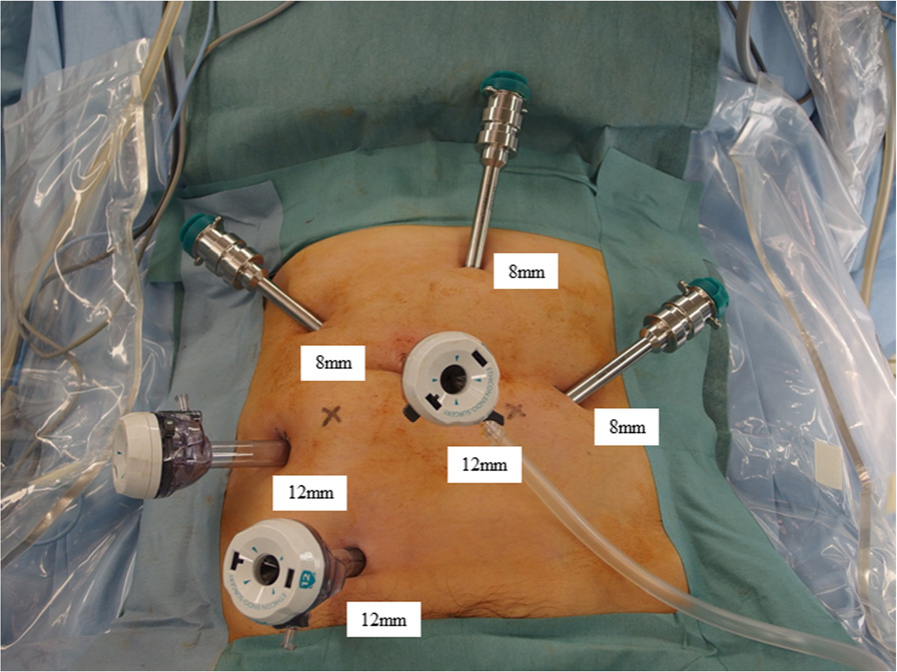
**Port placement for left colon cancer and rectal cancer.**

**Figure 2:**
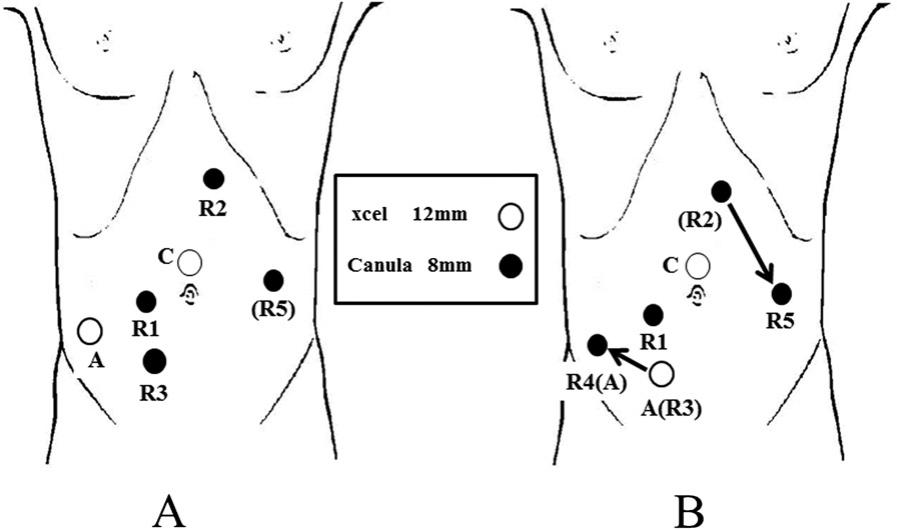
**Port placement. (A)** Port placement using the medial to lateral approach (lateral phase). **(B)** Port placement in the pelvic space (pelvic phase).

**Figure 3:**
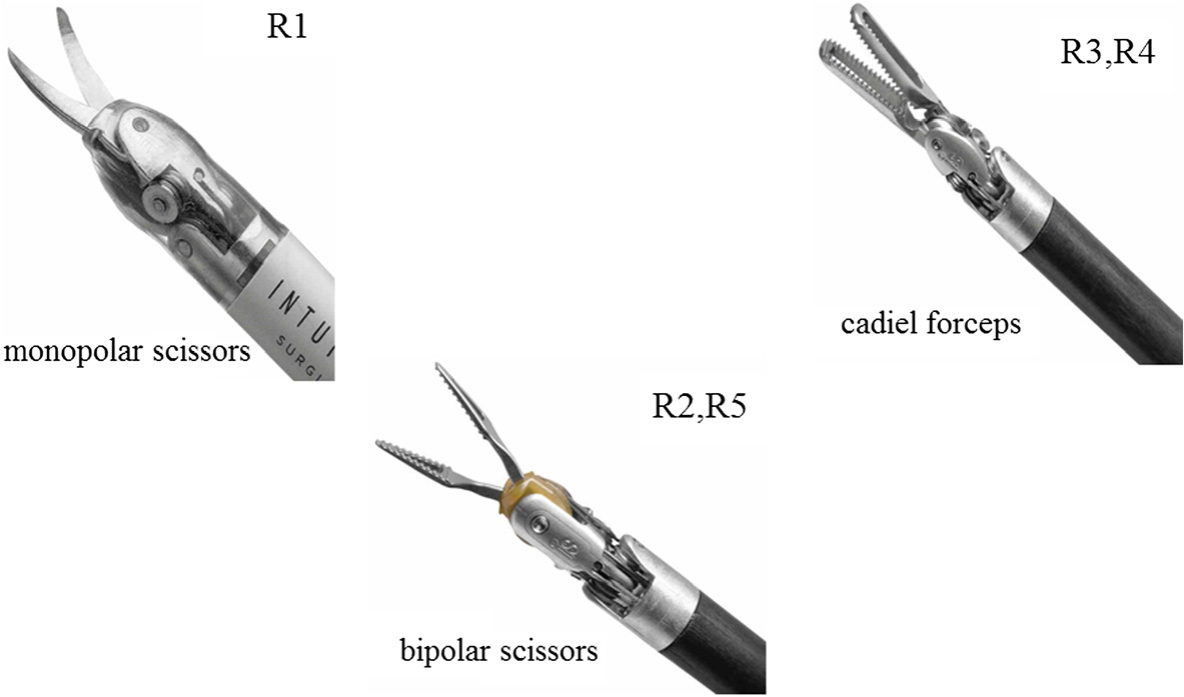
**Instruments of the da Vinci S-HD-assisted colorectal surgery device.**

The robot used in this study was the da Vinci S Surgical System (Intuitive Surgical, Sunnyvale, CA, USA). We usually use three 8-mm trocars (Intuitive Surgical, Sunnyvale, CA, USA) and three 12-mm trocars (Ethicon, Inc., Cincinnati, OH, USA) (Figure 1). The trocar placement doing the medial to lateral approach (Figure 2A; lateral phase) and during the procedure in the pelvic space (Figure 2B; pelvic phase) is presented in Figure 2. We usually use monopolar scissors (R1) and cadiere forceps (R3 or R4) for the right hand and bipolar scissors (R2 or R5) for the left hand (Figure 3). Our procedure for lymph node dissection includes left colic artery-preserving D3 to maintain a good blood supply on the oral side of the colon.

The operative procedures for sigmoid colon, recto-sigmoid, and upper rectal cancer were performed using a 3-cm skin incision in the umbilicus, followed by laparotomy. The specimen was then removed through the incision, and reconstruction was performed in the abdominal cavity under laparoscopy. On the other hand, the specimen was removed from the anal side in case of intersphincteric resection or abdominoperineal resection.

### Statistical analysis

All continuous variables are expressed as the median (range) and were compared using the Mann-Whitney test between the robotic surgery and conventional laparoscopic surgery groups. The chi-square test and Fisher’s exact test were used to compare discrete variables.

Moreover, in order to improve objectivity and approximate a randomized controlled study, we used the propensity score matching method to balance the observed covariates between the robotic surgery and conventional laparoscopic surgery groups. The propensity score matching method was employed to reduce treatment selection bias and potential confounding biases due to differences between the two treatment groups.

Propensity scores were calculated for each patient using a multivariable logistic regression analysis based on the following covariates: age, gender, American Society of Anesthesiologists (ASA) score, body mass index (BMI), operative history, tumor location, and TNM staging. Discrimination was evaluated using the c-statistics. This model yielded a c-statistics of 0.673 (95% CI, 0.515 to 0.830), indicating an appropriate ability to differentiate between patients who underwent robotic surgery and those who underwent conventional laparoscopic surgery. For the prediction model, the calibration was assessed according to the Hosmer-Lemeshow goodness-of-fit test, which showed excellent calibration (*P* = 0.863). One-to-two pair matching was performed between the two treatment groups, pairing the patients with the closest propensity scores (within 0.03 on a scale of 0 to 1). This matching was successful, as the c-statistics was well balanced (0.560, 95% CI, 0.340 to 0.780). Finally, we compared the ten robotic surgery patients with 20 matched conventional laparoscopic surgery patients with respect to short-term surgical outcomes.

All statistical analyses were performed using the SPSS Statistics software program (version 22; SPSS Inc., Chicago, IL, USA), with a *P* value of <0.05 being considered to indicate statistical significance.

## Results

### Patient characteristics

A total of 131 patients (ten robotic surgeries and 121 conventional laparoscopic surgeries) were included in this study. The patients in the robotic and conventional laparoscopic groups were comparable in terms of age, gender, American Society of Anesthesiologists (ASA) score, body mass index (BMI), operative history, tumor location, TNM staging, and the preoperative serum CEA level. All patients were matched as closely as possible in terms of their selection criteria and were well balanced (data not shown). However, in order to improve objectivity and approximate a randomized controlled study, we used the propensity score matching method. The data for both groups are shown in Table 1. The median age of the ten patients of the robotic surgery group was 64.5 years (range: 55 to 72), with that of the 20 patients of the conventional laparoscopic surgery group was 64.0 years (range: 48 to 79) (*P* = 0.947). Six of the ten robotic surgery patients were male, compared with 11 of the 20 laparoscopic patients (*P* = 1.000). The median BMI was 21.98 kg/m^2^ (range: 17.9 to 28.4) in the robotic surgery group compared with 24.1 kg/m^2^ (range: 15.0 to 29.7) in the conventional laparoscopic surgery group (*P* = 0.509). Two (20%) of the patients on the robotic surgery group and three (15%) of the patients in the conventional laparoscopic surgery group had a previous history of surgery (*P* = 1.000). There were no significant differences between the two groups in terms of the ASA score, tumor location, surgical stage, or preoperative serum CEA level (*P* = 0.666, 1.000, 0.931, and 0.758, respectively).

**Table 1:** **Patient demographic and clinical characteristics after propensity score matching**

	**Robotic**	**Laparoscopic**	***P*** **value**
	**(** ***N*** **= 10)**	**(** ***N*** **= 20)**	
Age (years)	64.5(55 to 72)	64.0(48 to 79)	0.947
Sex (male/female)	6/4	11/9	1.000
BMI (kg/m^2^)	21.98(17.9 to 28.4)	24.1(15.0 to 29.7)	0.509
Previous abdominal surgery	2	3	1.000
ASA score			0.666
1	1	3	
2	9	15	
3	0	2	
Location			1.000
Sigmoid colon	3	6	
Rectum	0	2	
Surgical cage			0.931
I	5	12	
II	3	4	
IIIa	1	3	
IIIb	1	1	
Preoperative serum CEA	3.0(1.1 to 31.3)	3.5(1.1 to 31.1)	0.758

### Clinicopathological outcomes

The clinicopathological variables are compared in Table 2. There were no surgical mortalities or patients requiring reintervention within 30 days in either group. The median operative length was significantly longer in the robotic surgery group (median: 417 min, range: 295 to 629 min) than in the conventional laparoscopic surgery group (median: 290 min, range: 210 to 452 min) (*P* = 0.001). There were also no significant differences in the amount of estimated blood loss between the robotic surgery group (median: 45.0 ml, range: 5 to 250 ml) and the conventional laparoscopic surgery group (median: 55.0 ml, range: 15 to 300 ml) (*P* = 0.658). No patients required conversion to open surgery in the robotic surgery group, while one patient (5.0%) in the conventional laparoscopic surgery group was converted to open surgery (*P* = 1.000). No case of reoperation due to complications was encountered in either group. There were no significant differences in the rate of postoperative complications (*P* = 0.584), and no operative mortality was observed in either group. There were also no significant differences regarding flatus passage after surgery between the robotic surgery group (median: 1 day, range: 1 to 4 days) and the conventional laparoscopic surgery group (median: 1 day, range: 1 to 3 days) (*P* = 0.644). In terms of the length of hospital stay, the median stay of 9.0 days observed in the robotic surgery group (range: 7 to 17 days) was not significantly shorter than that of 11.0 days noted in the conventional laparoscopic surgery group (range: 7 to 39 days) (*P* = 0.243). The median number of lymph nodes harvested was also not significantly different between the robotic surgery group (median: 14.0, range: 2 to 18) and the conventional laparoscopic surgery group (median: 13.0, range: 5 to 30) (*P* = 0.243).

**Table 2:** **Clinicopathological outcomes after propensity score matching**

	**Robotic**	**Laparoscopic**	***P*** **value**
	**(** ***N*** **= 10)**	**(** ***N*** **= 20)**	
Mean operating time (min)	417(295 to 629)	290(210 to 452)	0.001
Estimated blood loss (g)	45(5 to 250)	55(15 to 300)	0.658
Further surgery	0	0	
Postoperative complication	2	2	0.584
Anastomotic leakage		1	
Ileus	1		
Wound problem	1	1	
Flatus (days)	1(1 to 4)	1(1 to 3)	0.644
Postoperative hospital stay	9(7 to 17)	10.5(7 to 39)	0.243
Retrieved LN	14.0(2 to 18)	13.0(5 to 30)	0.243

## Discussion

The apparent advantages of robotic surgery include a three-dimensional view, the ability to use 7-degree-of-freedom forceps, the elimination of physiological tremors, and stable camera control.

In the urological field, robotic surgery has been accepted and evaluated with respect to both safety and efficacy [15,16]. In the field of colorectal surgery, the first robotic surgery was reported in 2002 [17]. Since then, robotic surgery has been gradually introduced into the setting of rectal surgery as a result of its superior movement and camera control in the narrow pelvic space. However, there is inadequate evidence in the literature regarding the feasibility and safety of this technique. Although we experienced only a small number of cases (ten cases) of robotic surgery for colorectal cancer, we compared the outcomes of conventional laparoscopic surgery (121 cases) using the propensity score matching method in order to approximate a randomized controlled study. To the best of our knowledge, our case-matched study is the first study to use the propensity score matching method to compare the outcomes of robotic and laparoscopic surgery in patients with colorectal cancer.

In this study, we compared various parameters between the robotic surgery group and the conventional laparoscopic surgery group. In order to evaluate the invasiveness of the procedures, we compared the operative length, amount of estimated blood loss, timing of flatus passage, and length of postoperative hospital stay. In our series, there were no significant differences in the amount of estimated blood loss, timing of flatus passage, or length of postoperative hospital stay between the robotic surgery group and the conventional laparoscopic surgery group. In terms of the operative length, the median 417 min observed in the robotic surgery group (range: 295 to 629 min) was significantly longer than the 290 min noted in the conventional laparoscopic surgery group (range: 210 to 452 min; *P* = 0.001). A longer operative time is one disadvantage of robotic surgery, in addition to the high cost, lack of tactile sensation, and narrow visual field, as compared with conventional laparoscopic surgery. However, we believe that the technique of robotic surgery will improve with experience after a learning curve. Indeed, previous studies have reported comparable operative times between robotic surgery and conventional laparoscopic surgery for rectal cancer [18,19].

With regard to the pathological findings, including tumor differentiation, tumor depth, node metastasis, lymphatic invasion, and vascular invasion, there were no significant differences between the groups (data not shown). In terms of the median number of harvested lymph nodes, there were no significant differences between the robotic surgery group (median: 14.0, range: 2 to 18) and the conventional laparoscopic surgery group (median: 13.0, range: 5 to 30; *P* = 0.243), as shown in Table 2. No patients had a positive distal resection margin or positive circumferential resection margin in either group.

The zero rate of conversion of robotic surgery for colorectal cancer observed in this study is excellent, although there were no significant differences between the robotic surgery group and the conventional laparoscopic surgery group. The rate of conversion of conventional laparoscopic surgery was 5.0%, which is low compared to the findings of other reports. Our series of robotic surgery for colorectal cancer (*N* = 10) included no case of conversion, and only two cases of surgical complications due to ileus and wound healing issues were observed (Table 2); there were no severe complications. Moreover, the postoperative recovery results, including the timing of flatus passage and the length of postoperative hospital stay, were similar between the two groups.

Our results demonstrated the feasibility and safety of performing robotic surgery for colorectal cancer with respect to perioperative outcomes.

This study was limited by its small sample size in the robotic surgery group. However, in order to improve objectivity and approximate a randomized controlled study, we used the propensity score matching method. The matching was successful, as the c-statistics revealed a well-balanced curve (0.535), indicating reliable results with respect to the finding that robotic surgery is equivalent to conventional laparoscopic surgery in patients with colorectal cancer regarding short-term outcomes, including perioperative and pathological results.

Moreover, this study provides an initial comparison between robotic surgery and conventional laparoscopic surgery for colorectal cancer which offers a foundation for larger randomized controlled studies.

## Conclusions

Our early experience indicates that robotic surgery is a feasible and safe procedure in patients with colorectal cancer. Although there were no significant benefits regarding the perioperative and oncological results, robotic surgery provides better outcomes, especially in patients undergoing rectal surgery. However, before extending the indications for this procedure, it is necessary to evaluate the perioperative and long-term oncological safety in large randomized controlled trials.

## Abbreviations

BMI: body mass index
ASA: American Society of Anesthesiologists
